# Do We Have to Shift Three Doses of Hepatitis B Vaccine Instead of Four Doses in Chronic Renal Failure: Think Before Action

**DOI:** 10.5812/hepatmon.8037

**Published:** 2013-03-13

**Authors:** Baris Afsar

**Affiliations:** 1 Department of Nephrology, Konya Numune State Hospital, Konya, Turkey

**Keywords:** Dialysis, Hepatitis B Vaccines

Dear Editor,

I read with interest the recently published article written by Ahmadi et al. in Hepatitis Monthly. The authors found that four doses of 40 μg did not lead to significantly more seroconversion than three doses of 20 μg in chronic renal failure patients (CKD) not on dialysis (1). Although the study is informative, some limitations of the study should be mentioned. First of all in the abstract, results section is somewhat confusing. The authors reported that differences in seroconversion rates after four doses of 40 μg (80.88%) compared with three doses of 20 μg (92%) were not significant (P = 0.4124). However in other sentence it is stated that seroconversion after four doses of 40 μg (80.8%) was also significantly higher than that after three doses of 40 μg (77%) (P = 0.004). Thus it is not clear from these sentences whether seroconversion is significant between these two groups. Secondly, although the authors calculate sample size as 137 patients, however the study included 51 patients. Thus the study raises concerns about the power of study to detect statistical difference between groups. In the methods section it is not clear whether there is any dropout. Although the authors reported that there were no withdrawals in patients section, the study began with 64 patients and ended with 51 patients. So it seems that 13 patients were lost during the study. The authors stated that glomerular filtration rate (GFR) was calculated by Cockcroft-Gault formula but this formula is not so valid for patients with chronic kidney disease (CKD) and MDRD formula would be better for calculation of GFR. It is also not clear whether the mean HBs Ab level after four doses of the 40 μg vaccine compared with three doses of the 20 μg vaccine was different or not. Although they did not reach statistical differences (182.2 ± 286.7 vs. 107.6 ± 192.1, P:0.3), in the first sentence it is concluded that HBs Ab level after four doses of the 40 μg vaccine in comparison with three doses of the 20 μg vaccine is higher and in the following sentence it was reported that the mean HBs Ab level after four doses of 40 μg (182.2 ± 286.7) was significantly higher than that attained after three doses of the 40 μg vaccine (96.9 ± 192.1) (P = 0.004). Lastly regarding the multivariate analysis the gender should be put in the model, since the two groups were different with respect to gender. Thus although the study is informative, various limitations of the study might preclude to reach firm conclusions. Further randomized and long lasting studies are needed whether three doses are as effective as four doses. Until then, 4 doses of vaccination will be suitable in these patients as suggested (2, 3).
